# News and Notes

**Published:** 1901-04

**Authors:** 


### *NEWS AND NOTES.*

---

A BOUNTY on rats is being paid by the Stockholm authorities as a precaution against bubonic plague.

---

THE Woman's Hospital has been incorporated in Los Angeles, California, with a capital stock of $20,000.

---

THE prevalence of scarlet fever in Waycross has called forth an ordinance forbidding public gatherings of all kinds until the endemic is checked.

---

THE State legislature of Alabama recently enacted a law prohibiting Christian scientists and faith-cure doctors from doing business in that State.

---

DR. WILLIAM OSLER, of Baltimore, has given $1,000 to the endowment fund of the library of the Maryland Medical and Chirurgical Faculty.

---

THE *Stylus* has been purchased by, and will be consolidated with, the *Interstate Medical Journal,* and the two publications continued under the latter name.

---

A BUST of Dr. Horace Green, a pioneer in the medical profession of the city of New York, was presented to the New York Academy of Medicine on February 7.

---

GRADUATES of the Bellevue Hospital and of the New York University Medical College have organized at Chicago a western alumni association of the two institutions named.

A DESPATCH to the London *Chronicle* from Vienna states on the authority of a telegram from Athens that the grave of Hippocrates was discovered during excavations at Larissa. A royal commission was immediately sent to verify the report.

---

THE Western Ophthalmologic and Oto-Laryngologic Association will meet in its next annual session in Cincinnati, Ohio, April 11 and 12. A fine program has been arranged, and the medical profession is cordially invited to attend the sessions.

---

THE total number of students in the universities of Germany in the current winter semester is 34,363, as against 34,389 last summer, and 33,353 in the previous winter. The number of students of medicine shows a considerable decrease, being 7,113 as against 7,543 last winter.

---

THE Italian government has tendered Dr. Eugene Wasdin, of the United States Marine Hospital Service, the Cross of Officer of the S. S. Maurizio et Lazzaro, in recognition of his services in verifying and confirming the Italian studies and discoveries regarding the nature of yellow fever.

---

A MAN was severely injured by being dragged for a considerable distance by a trolly car. On making an examination, the ambulance surgeon of Bellevue Hospital expressed the opinion that because the pupil of the right eye was somewhat more dilated than that of the left, there was concussion of the brain present. "O doctor," said the patient, "you needn't mind the appearance of that eye. It's a glass one."

---

ACCORDING to the *New York Medical Journal*, the health department of Watertown, N. Y., has placed on exhibition a man suffering from smallpox, the health department taking that remarkable method to convince skeptical physicians and others that the cases of smallpox in town are actually that disease. The man afflicted was placed on a couch before a large window at his home, which faces directly upon the sidewalk.

---

ON March 9th a mob of about three hundred men, mostly Italians, attempted to burn a small frame isolation hospital in course of construction by the Board of Health at Orange, N. J., and it was necessary to invoke the assistance of the fire and police departments to put out the flames and protect the premises. The hospital is designed for the accommodation of smallpox patients, and there are at present two cases of the disease in the care of the health authorities.

---

TWENTY-SEVEN years ago, says the *Medical Record*, Dr. Rudolph Herzog, of Tübingen, undertook excavations in the island of Cos, with a view of finding the temple of Æsculapius. At a depth of 80 centimeters (32 inches) he came upon a mosaic flooring which represented Orpheus charming the wild beasts. At a depth of 2.5 meters (nearly eight feet), in the neighborhood of the church of Saint Anna, he found two columns, and not far from them the remains of an aqueduct and a small statue of a young man. Great importance is attached to Dr. Herzog's discovery of the supposed temple of Æsculapius. The excavations are still in progress, and it is hoped that many antiquities will be unearthed.

---

THE Colorado State Medical Society offers a prize of $25 for the best essay, if deemed worthy of the prize, pointing out the dangers to public health and morals, especially to young persons, from quackery as promulgated by public advertisements. The competition is open to all. Essays must be typewritten, in the English language, and submitted before May 15, 1901. Each essay must be designated by a motto and accompanied by a sealed envelope bearing the same motto, and inclosing the name and address of the author. The essay receiving the prize will become the property of the society for publication; others will be returned on application. Essays should be sent to the Literature Committee, Room 315, McPhee Building, Denver, Colorado.

---

THE *Galveston News* of a late date reports the adoption of a resolution by the Democratic Convention at Waco, on December 22, as follows: "Resolved, that we favor the creation of a State board of health with the provision for the collection of vital statistics of the State, as provided for by the constitution, article 16, section 32, and the enactment of laws to distribute the expenses of improvement of quarantine equitably between the State, counties, and municipalities." The Texas State Medical Association has appointed a committee to draft a bill creating and defining the powers of a State board of health as contemplated by the resolution. Its scope is expected to enlarge and make more efficient the State Health Department than it has ever been heretofore.

---

AN amusing incident occurred last week in a large apartment house in the borough of the Bronx, which had been quarantined on account of a case of smallpox. While the inspectors of the Board of Health were making a vaccinating tour of the premises they came across a sneak-thief in one of the flats who, not appalled by the danger of infection, had taken advantage of the confusion of the occasion to slip in and help himself to such valuables as could conveniently be appropriated. He represented, however, that he was "the son of the lady of the house," and, at the request of the inspectors, obligingly offered his arm for vaccination. Soon afterward "the lady of the house" herself appeared, and as she promptly stated that she had no son, the fellow was arrested, when a number of rings and other pieces of jewelry were found in his pockets.

